# Assessment of a trap based *Aedes aegypti* surveillance program using mathematical modeling

**DOI:** 10.1371/journal.pone.0190673

**Published:** 2018-01-05

**Authors:** Raquel Martins Lana, Maíra Moreira Morais, Tiago França Melo de Lima, Tiago Garcia de Senna Carneiro, Lucas Martins Stolerman, Jefferson Pereira Caldas dos Santos, José Joaquín Carvajal Cortés, Álvaro Eduardo Eiras, Cláudia Torres Codeço

**Affiliations:** 1 Programa de Computação Científica, (PROCC), Fundação Oswaldo Cruz (Fiocruz), Rio de Janeiro, Rio de Janeiro, Brazil; 2 Centro Universitário de Belo Horizonte (UniBH), Belo Horizonte, Minas Gerais, Brazil; 3 Departamento de Computação e Sistemas (DECSI), Instituto de Ciências Exatas e Aplicadas (ICEA), Universidade Federal de Ouro Preto (UFOP), João Monlevade, Minas Gerais, Brazil; 4 Departamento de Computação, Universidade Federal de Ouro Preto, Ouro Preto, Minas Gerais, Brazil; 5 Programa de Pós-Graduação em Epidemiologia em Saúde Pública, Escola Nacional de Saúde Pública Sérgio Arouca (ENSP), Fundação Oswaldo Cruz (Fiocruz), Rio de Janeiro, Rio de Janeiro, Brazil; 6 Laboratório de Doenças Parasitárias, Instituto Oswaldo Cruz/Fiocruz, Rio de Janeiro, Rio de Janeiro, Brazil; 7 Laboratório de Ecologia Química de Insetos Vetores (Labeq), Departamento de Parasitologia Instituto de Ciências Biológicas (ICB), Universidade Federal de Minas Gerais, Belo Horizonte, Minas Gerais, Brazil; Centro de Pesquisas René Rachou, BRAZIL

## Abstract

The goal of this study was to assess the goodness-of-fit of theoretical models of population dynamics of *Aedes aegypti* to trap data collected by a long term entomological surveillance program. The carrying capacity *K* of this vector was estimated at city and neighborhood level. Adult mosquito abundance was measured via adults collected weekly by a network of sticky traps (Mosquitraps) from January 2008 to December 2011 in Vitória, Espírito Santo, Brazil. *K* was the only free parameter estimated by the model. At the city level, the model with temperature as a driver captured the seasonal pattern of mosquito abundance. At the local level, we observed a spatial heterogeneity in the estimated carrying capacity between neighborhoods, weakly associated with environmental variables related to poor infrastructure. Model goodness-of-fit was influenced by the number of sticky traps, and suggests a minimum of 16 traps at the neighborhood level for surveillance.

## Introduction

Arthropod-borne viruses are responsible for a high disease burden worldwide [1, 2]. Many arboviruses originally evolved and diversified in the tropics and currently show increasing virulence and invasive characteristics associated with abrupt and explosive outbreaks, even in temperate regions [3]. The main arthropod vectors involved in viral transmission to humans are ticks, sandflies and mosquitoes. Mosquitoes of the genus *Aedes* are among the most studied vectors due to their role on the transmission of several arboviruses with significant public health impact, including yellow fever, dengue, zika (Flaviviridae, Flavivirus) and chikungunya (Togaviridae, Alphavirus) [4].

The abundance of *Aedes aegypti* in a territory is an important risk factor for the emergence and maintenance of these diseases [4], and many countries spend a large amount of resources on vector surveillance and transmission control measures. In Brazil as well as other countries that follow the World Health Organization (WHO) guidelines, the standard surveillance protocol is the household survey, which generates regular estimates of the Premise Index (PI), with values above 4% indicating risk of dengue outbreak [5]. However, many studies have suggested that such larval indices are not sensitive or efficient for monitoring the female adult *Aedes* population, which is the mosquito subpopulation directly linked to virus transmission [6, 7].

A trap-based surveillance program for continuous estimation of female adult *Ae. aegypti* is an alternative to household surveys. Traps are less intrusive, require less labor, and can achieve better spatial coverage and temporal resolution [8]. There are some initiatives to use trap based surveillance worldwide, using different traps and protocols, but no consensus has emerged yet on the best approach [6].

In Brazil, several cities have started trap-based *Aedes* surveillance initiatives in the last decade, in a quest for new entomological indices to guide their dengue control activities. The city of Vitória, capital of Espírito Santo State, is one of them. The city has been the scene of dengue epidemics since 1995 when DENV-2 serotype arrived [9]. Currently, all four dengue serotypes circulate in the city, with dominance of DENV-1 and DENV-4 since 2013 [10]. In 2016, the region witnessed the arrival of Zika (2,276 cases) and Chikungunya (313 cases) [11].

Vitoria’s trap surveillance program, named “Intelligent Dengue Monitoring System” (MI-Dengue) (Ecovec SA, Belo Horizonte, Brazil) employs sticky traps (MosquiTRAP) baited with synthetic oviposition attractant to capture gravid *Aedes* mosquitoes [12]. Captured mosquitoes are identified and counted in the field and data are sent immediately to a data center via cell phone [13, 14]. The Mean Female *Aedes sp*. Index *IMFA* is calculated as the ratio between the number of mosquitoes and the number of traps in a given area and mapped to inform the city’s vector control crew on the location of high infestation neighborhoods that will be targets for intervention, which includes source reduction and adulticide application [5]. Previous studies have investigated the adequacy of the MI-Dengue system in other cities in terms of effectiveness in reducing dengue cases [13, 14] and its cost-effectiveness [14]. Some studies suggest that the MosquiTrap is less sensitive than other traps to monitor the seasonal dynamics of *Ae. aegypti* [6, 15].

Climate and landscape factors are important determinants of *Ae. aegypti* abundance. Vitória city is a humid climate city located in the Brazilian coastline, where seasonal effects are mostly temperature-driven [6, 16]. In a meta-analysis, Couret and Benedict [17] concluded that temperature is a sufficient factor to explain variation in the development rate of *Ae. aegypti*. Among landscape factors, the availability and quality of breeding sites determine the carrying capacity of a given area. Intraspecific competition within breeding sites affect the mortality rate of larvae and consequently the productivity of an area [18, 19].

Environmental carrying capacity is the maximum population load an area can support [20] and for *Ae. aegypti*, it should increase with the amount of suitable breeding sites. Mathematically, the carrying capacity is a prominent modeling feature in the logistic function as a factor controlling the growth of the population. An assessment of the carrying capacity would be useful for informing control activities, particularly, if mechanical control is to be used. Although the carrying capacity is not directly observable, it can be estimated from abundance data using a mathematical model describing its population dynamics. These models take the form of a set of functions mechanistically describing the relationship between the observed variable (number of mosquitoes trapped per week) and the factors regulating the mosquito life parameters.

In a previous work, we estimated the carrying capacity of *Ae. aegypti* in neighborhoods of Rio de Janeiro using ovitrap data [21]. The model has four equations, describing the dynamics of egg, larvae, pupae and adults. The carrying capacity *K* is assumed to be constant and control the maximum load of the egg compartment. Temperature is the external variable that controls the seasonality of the observed mosquito abundance. In the present study, the same mathematical model is used to estimate the carrying capacity of *Ae. aegypti* in the city of Vitoria as a whole and in each of its neighborhoods separately. In particular, we investigate if the estimated carrying capacity is associated with differences in human density and environmental variables related to the presence of breeding sites. Finally, we discuss the potential inclusion of model-based carrying capacity estimation in the surveillance routines.

## Methods

### Study area

**Fig 1:**
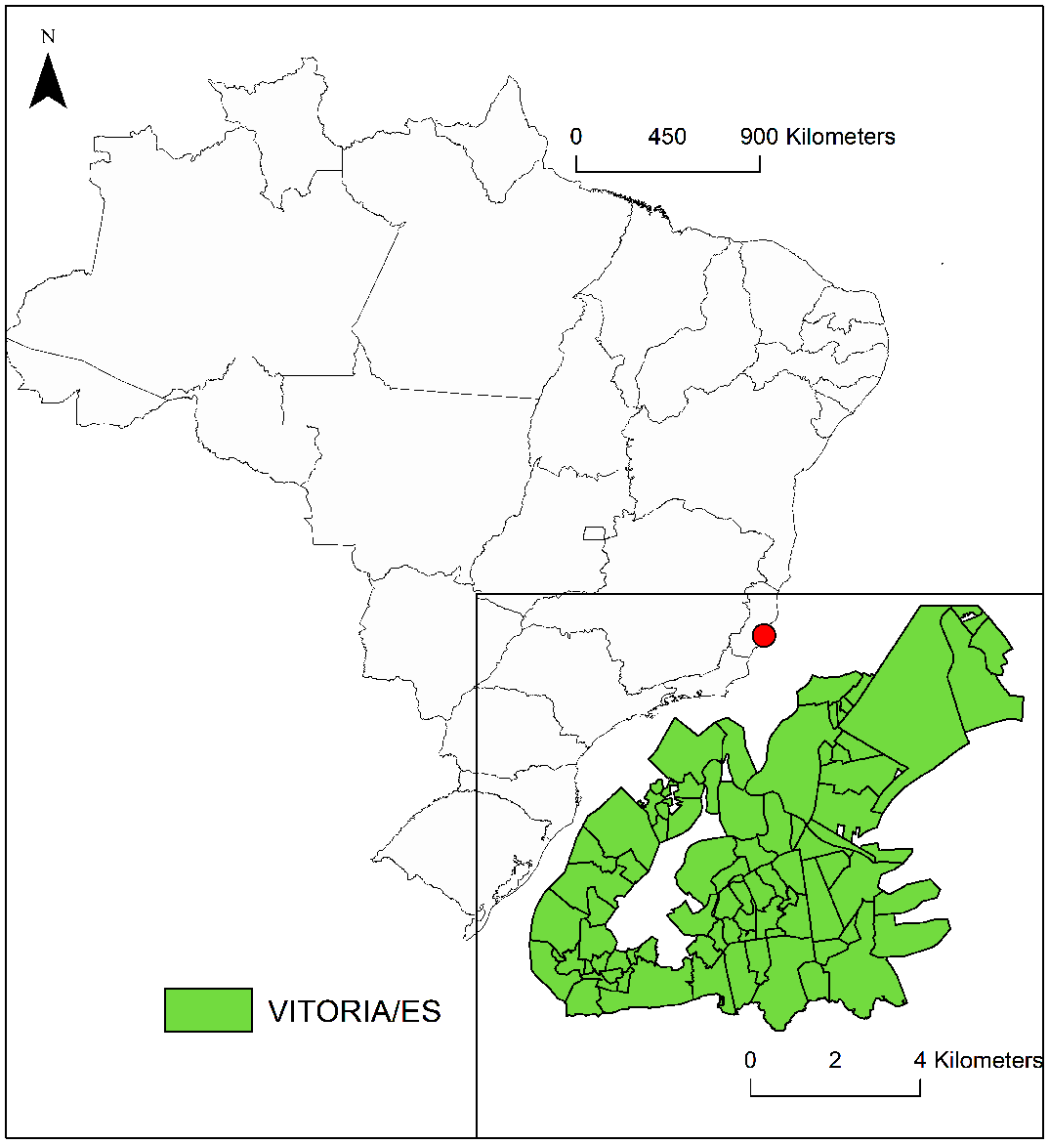
Vitória city and its neighborhoods, Espírito Santo, Brazil. The underlying shapefiles with political boundaries of Brazilian states and Vitória municipality are publicly and freely available at *Instituto Brasileiro de Geografia e Estatística* (IBGE, Brazilian Institute of Geography and Statistics) website http://downloads.ibge.gov.br/downloads_geociencias.htm.

Vitória (20°19’15” S, 40°20’10” W) is the capital of the state of Espírito Santo in southeastern Brazil (Fig 1). The city is located on a small riverine island, with altitudes ranging from 0 to 149 m. Its total population (355,875 inhabitants) lives in a 97.4*km*^2^ area, resulting in a 3,338.3/*km*^2^ population density [22]. The climate in Vitória is characterized as humid tropical, with average rainfall of 1,153 mm/year and an average temperature of 34.4°C in the summer and 24.4°C during the winter. The city is divided in a total of 80 neighborhoods [22], including slums with poor infrastructure and middle/high income areas with increasing degrees of urbanization and improved socioeconomic conditions.

### Data

#### Temperature time series

Images from the MODIS satellite (surface temperature sensor with 1000 meters of resolution) were obtained from the International Research Institute for Climate and Society (IRI) platform at the Columbia University Land Institute [23], for the period between January 2008 and December 2011, with a temporal resolution of 8 days. The satellite’s diurnal measurements are proxies for the daily maximum temperature. Good quality images (without cloud coverage) were selected and interpolated by the empirical Bayesian method [24]. A time series of maximum temperature with a time resolution of 8 days was obtained for each neighborhood by averaging the image values within the neighborhood geographical polygon. A daily time series of maximum temperature was created by linear interpolation using *na.approx* function from library *zoo* [25], R environment [26].

The mean daily temperature time series used in this work were calculated by fitting a regression model of the form

$$\begin{array}{c} Temp_{med}\left(t\right)=aTemp_{max}+b \end{array}$$

to Goiabeiras Automatic Station (20.3156°S, 40.3172°W) [27], where both mean and maximum temperatures were available (the former from a meteorological station, the latter from the satellite). The resulting expression was used as an approximation of the mean temperature in all remaining neighborhoods. On average, the mean temperature is five degrees below the maximum temperature in Vitoria. For details on temperature data, we refer the reader to table S1 Table.

#### Mosquito surveillance data

**Fig 2:**
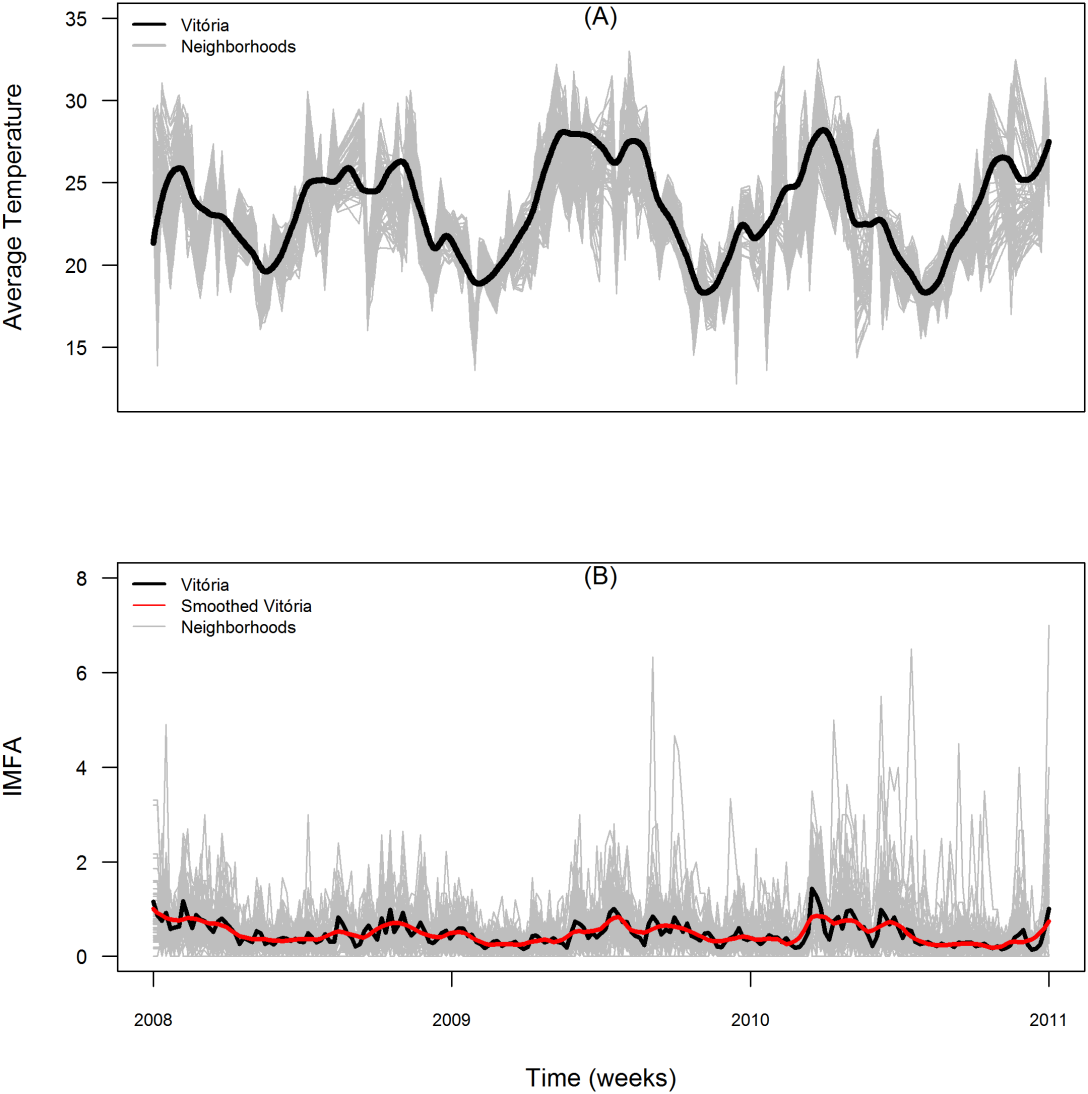
(A) Time series of diurnal surface temperature in Vitória city (black) and its neighborhoods (grey); (B) Mosquito infestation index *IMFA* (black: true values, red: smoothed curve) at city level and in each neighborhood (grey). The total period ranges from 1^st^ January 2008 to 31^th^ December 2011.

The entomological data consisted of a weekly time series with the number of captured *Ae. aegypti* mosquitoes, using sticky traps (called MosquiTraps) as part of the city’s entomological surveillance plan. Data from January 2008 to December 2011 were obtained from Ecovec SA (Belo Horizonte, Brazil). Traps were distributed on a regular grid with 250 *m* spacing covering the city and were inspected by trained personnel. The weekly entomological index (*IMFA*, Mean Female *Aedes* sp. index), calculated as the ratio between the total number of *Ae. aegypti* females captured and the number of inspected traps (Fig 2). The *IMFA* indexes were calculated for the city as a whole and for each neighborhood [13]. A total of 75 neighborhoods monitored by DENGUE-MI were used in the analysis. Some neighborhoods were grouped into Junctions due to difficulty in determining the exact location of some traps: Junction 1—Ariovaldo Favalessa, Alagoano and Morro Alagoano; Junction 2—Bela Vista and Nossa Senhora Aparecida; Junction 3—Praia do Suá, Morro de Santa Helena, Morro do Suá and Morro do São João; Junction 4—São Pedro, São José e Santos Reis; Junction 5—Segurança do Lar and Solon Borges. All *IMFA* data used in this work is available in S1 Table.

#### Neighborhood data

For each neighborhood, mosquito infestation was related to the following descriptor variables from the last Brazilian Census [22]: population and household count, area, population density, percentage of households with illegal energy supply, percentage of households in unpaved streets, closeness to garbage areas, without manhole and open sewage. These variables may potentially affect the carrying capacity of the *Ae. aegypti* population. Maps were created using polygons from the same source.

### Mathematical model

Our mathematical model describes a temperature-driven population dynamics of *Ae. aegypti* (introduced in [21]) and a new equation modeling the capturing process of adult mosquitoes by the trap network. In this section we introduce both model equations and temperature-dependent life-stage parameters.

#### Population dynamics

Eqs 1–4 describe the dynamics of eggs, larvae, pupae and adult populations of *Ae. aegypti*, respectively.

$$\begin{array}{c} \frac{\text{d}E}{\text{d}t}=\sigma_{0}A\;[1-\frac{E}{K}]-[\sigma_{1}+\mu_{1}]\;E \end{array}$$ (1)

$$\begin{array}{c} \frac{\text{d}L}{\text{d}t}=\sigma_{1}E-[\sigma_{2}+\mu_{2}]\;L \end{array}$$ (2)

$$\begin{array}{c} \frac{\text{d}P}{\text{d}t}=\sigma_{2}L-[\sigma_{3}+\mu_{3}]\;P \end{array}$$ (3)

$$\begin{array}{c} \frac{\text{d}A}{\text{d}t}=\sigma_{3}P-\mu_{4}A-\;[\alpha \frac{T_{n}}{H_{n}}A] \end{array}$$ (4)

$$\begin{array}{c} \frac{\text{d}Trapped}{\text{d}t}=\alpha \frac{T_{n}}{H_{n}}A \end{array}$$ (5)

The first equation describes the dynamics of the egg stock (*E*), with a density-dependent oviposition rate, which is regulated by the carrying capacity *K*, and a hatching rate *σ*_1_. Larvae (*L*) develop into pupae at a rate *σ*_2_, and pupae (*P*) into adults (*A*) at a rate *σ*_3_. Mortality at each developmental stage is described by the parameters *μ*_1_, *μ*_2_, *μ*_3_ and *μ*_4_. For details on this mathematical model, we refer the reader to [21].

The developmental rates *σ*_*i*_ are temperature-dependent according to the expression [28]

$$\begin{array}{c} \sigma_{i}\left(T\right)=\frac{\rho_{i}\frac{T}{298}\exp\;[\frac{a_{i}}{R}\;(\frac{1}{298}-\frac{1}{T})]}{1+\exp\;[\frac{b_{i}}{R}\;(\frac{1}{\tau_{i}}-\frac{1}{T})]} \end{array}$$ (6)

where *i* = 1, 2 and 3 in our model and *T* represents the mean temperature in Kelvin. For each life-history stage *i*, *ρ*_*i*_ represents the development rate at 298 *Kelvin* assuming no enzyme inactivation and the pairs {*a*_*i*_, *b*_*i*_} are specific parameters at 298 *Kelvin*, which are given by Focks et al. [28]. *τ*_*i*_ (usually denoted by *T*_1/2H_ in the literature) represents the temperature when half of the enzyme is deactivated by being subjected to high temperatures. Finally, *R* = 1.987 cal *K*^−1^ mol^−1^ is the universal gas constant [29].

#### Capturing process

The capturing process is modeled by Eq 5 where the variable *Trapped*(*t*) represents the total number of mosquitoes captured during the surveillance period until the instant *t*. The capture rate is given by *αT*_*n*_/*H*_*n*_, where *α* is the trap attractiveness (proportion of mosquitoes within a household attracted by the trap), *T*_*n*_ and *H*_*n*_ represents the number of traps and households in neighborhood *n*, respectively. The ratio *T*_*n*_/*H*_*n*_ is therefore a density of traps per household.

For calculating a theoretical *IMFA* in a week *w* based on our model, we use the equation

$$\begin{array}{c} imfa_{model}\left(w\right)=\frac{Trapped(w)-Trapped(w-1)}{T_{n}} \end{array}$$ (7)

### Model parameterization

All symbols and their meanings are listed in Table 1. The different life history parameters were obtained from the literature. The number of traps (*T*_*n*_), and households (*H*_*n*_) were parameterized according to each surveillance area. The attractiveness of the Mosquitrap, *α*, is not known a priori. However, previous field study described in Resende et al. [30] compared the catching rate of 1, 2, 4, 8, 16 traps placed in a single house with no evidence of ever exhausting the local mosquito population. This result suggests that the capture rate of a single trap is low. Here, we arbitrarily set *α* = 0.2 implying that 20% of the mosquitoes within a household would be captured per day. The impact of this choice is discussed later.

**Table 1:** Model parameters and values.

Parameters	Values	Sources
Oviposition rate (*σ*_0_)	1.0*day*^−1^	[31]
Egg eclosion rate (*σ*_1_)	*ρ*_1_ = 0.24, *a*_1_ = 10798, *b*_1_ = 100000, *τ*_1_ = 14184	[28] and [29]
Pupation rate (*σ*_2_)	*ρ*_2_ = 0.2088, *a*_2_ = 26018, *b*_2_ = 55990, *τ*_2_ = 304.6	
Emergence rate (*σ*_3_)	*ρ*_3_ = 0.384, *a*_3_ = 14931, *b*_3_ = −472379, *τ*_3_ = 148	
Egg mortality rate (*μ*_1_) Larva mortality rate (*μ*_2_) Pupa mortality rate (*μ*_3_) Adult mortality rate (*μ*_4_)	1/100*day*^−1^ 1/3*day*^−1^ 1/70*day*^−1^ 1/17.5*day*^−1^	[31]
Carrying capacity (*K*)	Fitted	
Trap attractiveness (*α*) Traps per neighborhood (*T*_*n*_) Households per neighborhood (*H*_*n*_)	0.2*day*^−1^ variable variable	ECOVEC IBGE (2010)

#### Model fitting

To estimate the carrying capacity *K* for each neighborhood as well as Vitória city as a whole, the following steps were taken: first, weekly time series of *IMFA* (*imfa*_*obs*_) were computed using either all city traps or the subset of traps within a neighborhood; second, *imfa*_*obs*_ was smoothed using local Polynomial Regression (degree 2, degree of smoothing = 0.08); and third step was to fit the mathematical model to *imfa*_*obs*_. For each candidate value for *K*, the model was numerically solved until its steady state using the temperature time series as a forcing function and the resulting time series of mosquito captures, *imfa*_*model*_, was calculated using 7.

The model was implemented in R [26] and calibrated using the *optimize* and the *FME∷modCost* functions to find the value of *K* that minimizes the mean squared error (MSE) between *imfa*_*mod*_ (at steady state) and *imfa*_*obs*_ [32].

## Results

### At the city scale

The city of Vitória was monitored with 1410 traps. The estimated carrying capacity for Vitória city as a whole was 2401 eggs (error = 3.55). This number should not be interpreted as an absolute measure of the maximum load of eggs, because this calculation is conditioned on the trap attractiveness (set at *α* = 0.2) and trap density. Later in the text, a formula is provided for calculating *K* using other values of *α*.

**Fig 3:**
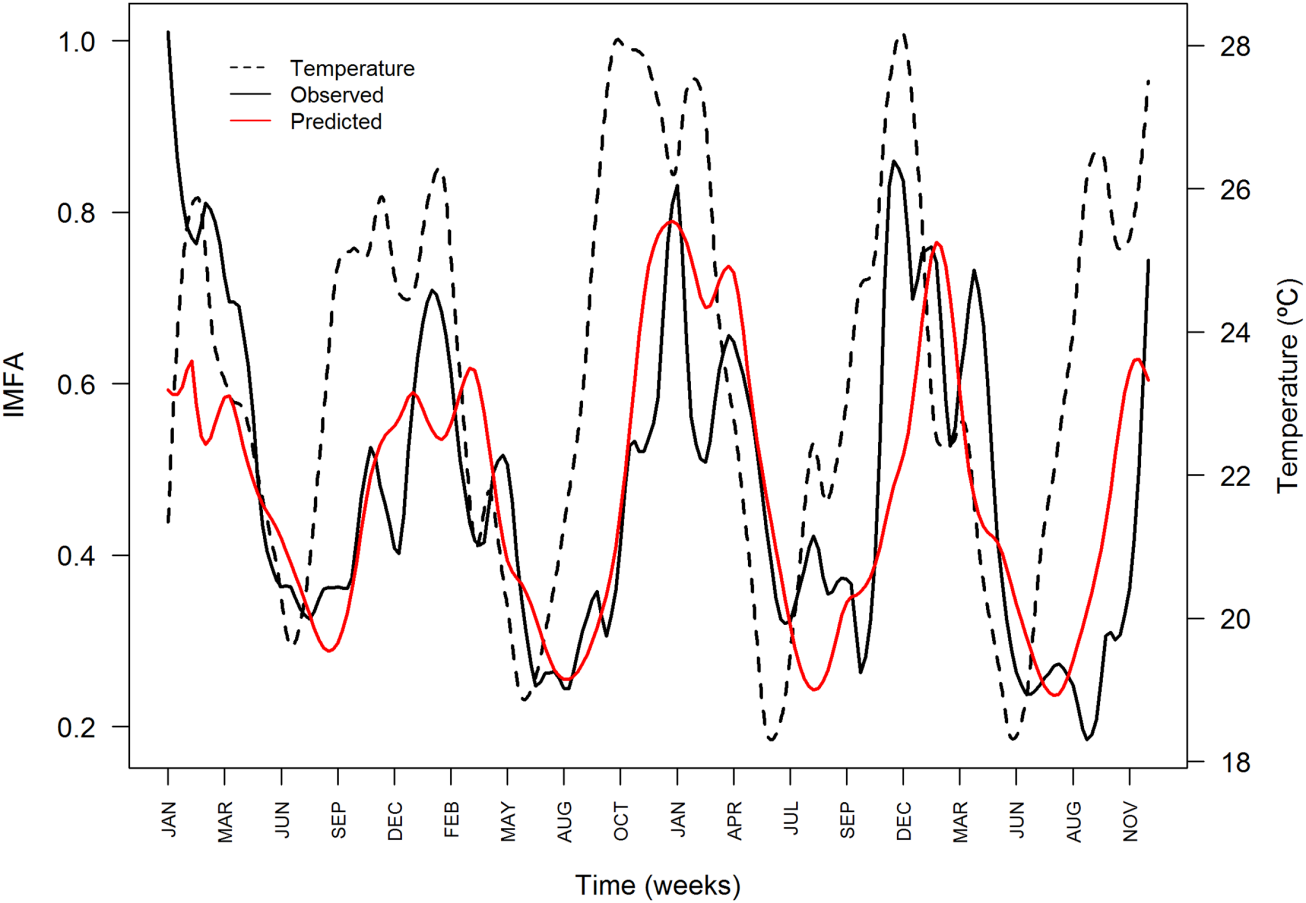
Time series of observed (black) and predicted (red) *Aedes aegypti* abundance index *IMFA* in Vitória, ES, from 1^st^ January 2008 to 31^th^ December 2011. Average temperature series is shown in dashed line.

Fig 3 compares the observed and predicted time series of *IMFA*. The model fitted well the seasonal fluctuations of *IMFA*, which tends to increase in October and peaks in December-January. The lowest abundance is observed from June to July.

### At the neighborhood scale

The 75 neighborhoods in Vitória vary considerably in area, population density, and infrastructure (Table 2). On average, a neighborhood has 4200 inhabitants, ranging from 98 to more than 39 thousand. There is still a large proportion of households located in streets with poor drainage, unpaved streets, and close to open garbage dumps.

**Table 2:** Association between demographic, environmental and entomological variables with *aIMFA* (average *IMFA* per neighborhood) and the estimated carrying capacity *K* of the neighborhoods of Vitoria city. Linear regressions were fitted (see text for details). Three asterisks indicate *p* − *value* < 0.05, two asterisks indicate *p* − *value* < 0.1.

Descriptors	Range	*aIMFA*	*K*
*aIMFA*	[0.2561,0.7712]		***
*K*	[1383,4143]	***	
Number of traps	[2,149]		
log(Number of inhabitants)	[4.5,10.5]		
Number of households	[27,14451]		
Area (*km*^2^)	[0.045,3]	**	***
Population density (*person*/*km*^2^)	[447,52479]		
% households with illegal energy supply	[0,30]		
% households with garbage in the streets	[0,8]	***	***
% households in unpaved streets	[0,20]		
% households with open sewage	[36,54]	**	
% households in streets without manhole	[0,79]		***

The number of traps installed per neighborhood varied from 2 in Junction 1, to 149, in Jardim Camburi. The mean number of traps was 18.67 with a median = 13 traps. The sampling effort increased linearly with the neighborhood area (*km*^2^), according to the equation *Traps* = 4.7 + 22.8 × *area* (*R*^2^ = 0.49).

**Fig 4:**
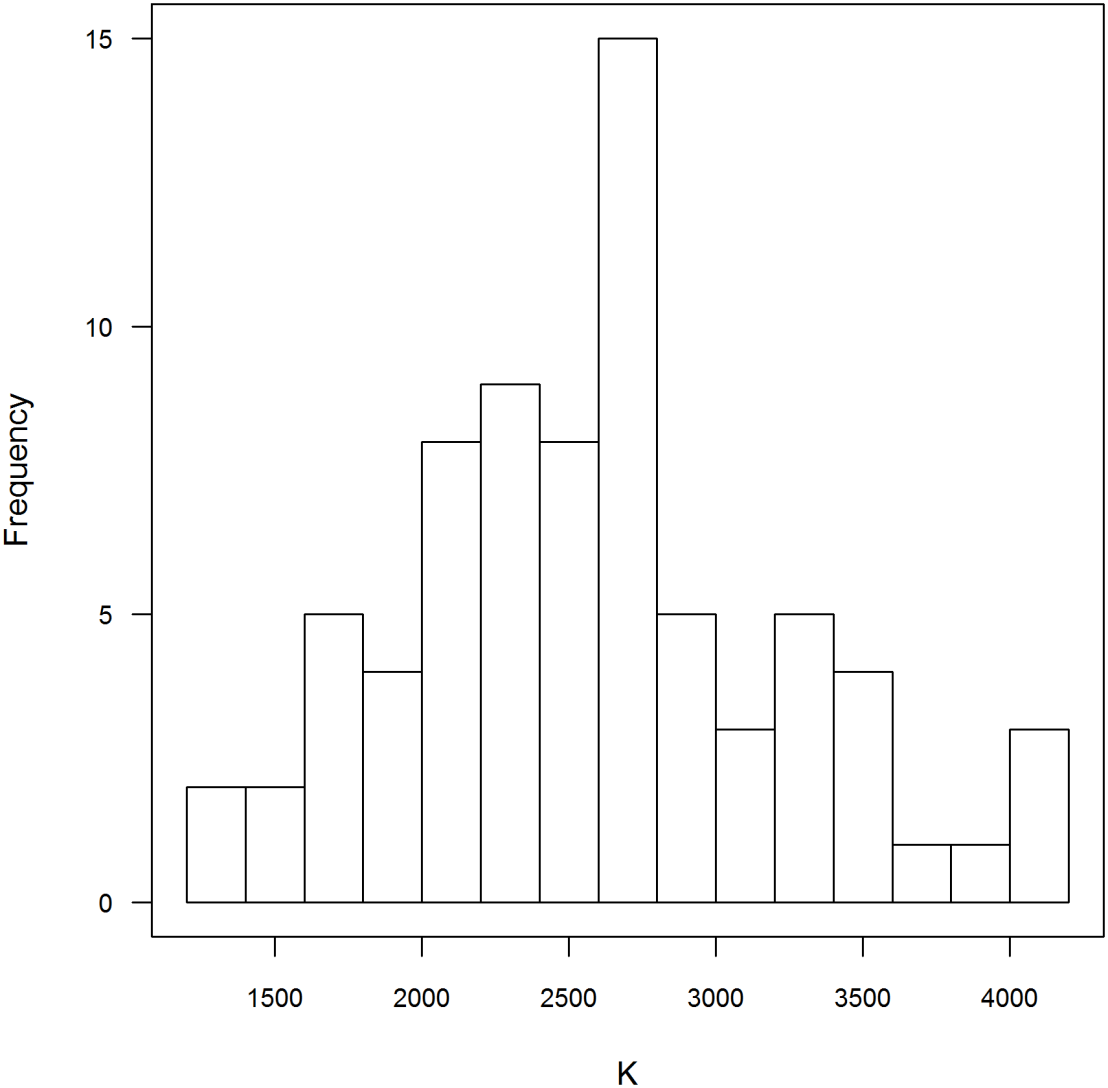
Frequency distribution of *Aedes aegypti* carrying capacity in Vitória neighborhoods.

The mathematical model was fitted independently to each of the 75 time series of *IMFA* from the neighborhoods of Vitória. Estimated *K* varied from 1383 to 4143. The distribution of *K* values are approximately normally distributed (Fig 4) with mean 2585 and standard deviation 649. The value of *K* estimated for Vitoria city (*K* = 2401) is lower than the average *K* estimated at the neighborhood level. Estimated *K* and average *IMFA* per neighborhood, *aIMFA*, are linearly associated (Table 2) as expected from the Eq 8 described below.

The mathematical model did not fit equally well to all time series. The median *MSE* was 8.8, with an interquantile range = [6.47, 13.18]. Four neighborhoods presented *MSE* > 30, two of them with few traps installed (2 and 3, respectively).

**Fig 5:**
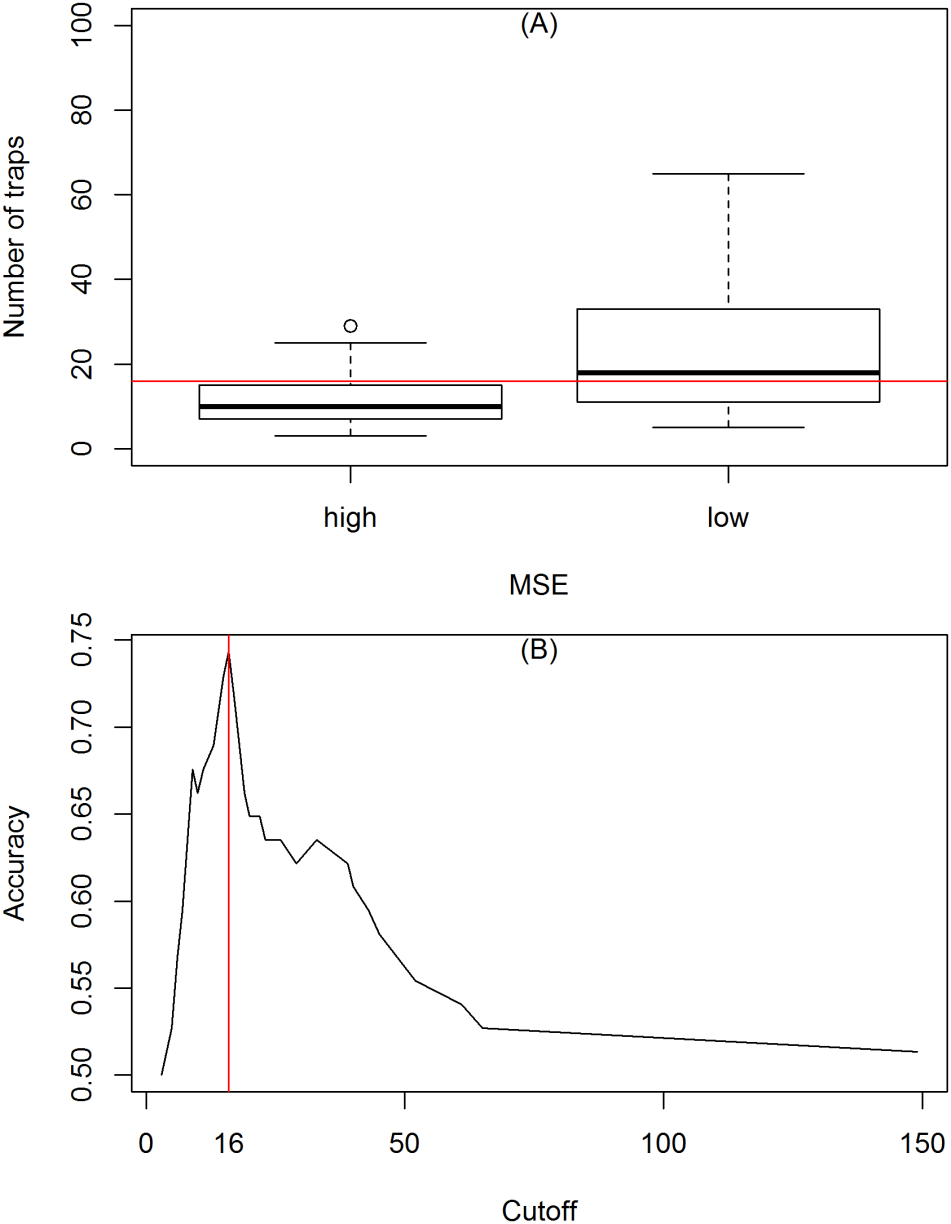
(A) Comparison between the number of traps used in the entomological surveillance resulted in high (poor fit) or low *MSE* (good fit). (B) ROC curve showing the number of traps that separating good fits from poor fits. The red line indicates the n = 16 traps that separates the two groups.

We further investigated if the goodness-of-fit was associated with the number of traps. The quality of the fit in each neighborhood was classified as below and above the average of *MSE*. Using ROC [33], we calculated as n = 16, the number of traps that best discriminated between the two groups (Fig 5). The poorest fits were observed in neighborhoods monitored with less than 10 traps.

**Fig 6:**
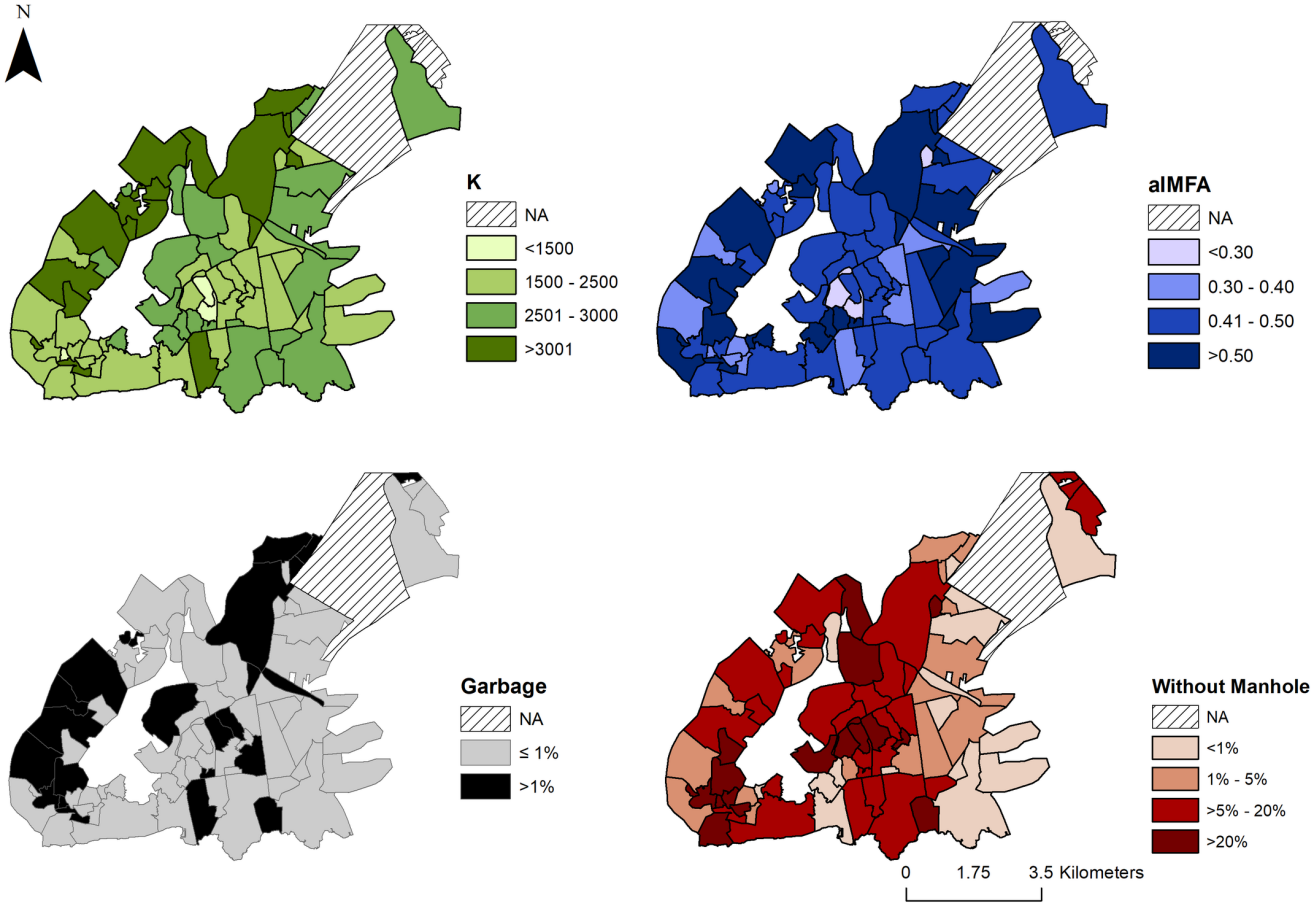
Maps of Vitória, ES, neighborhoods. Maps show estimated carrying capacity *K* (green), the average entomological index *aIMFA* (blue), *% households with garbage in the streets* (black) and *% households in streets without manhole* (red), respectively. The underlying shapefile with Vitória municipality and neighborhoods used to build this figure is publicly available and free to use at *Instituto Brasileiro de Geografia e Estatística* (IBGE, Brazilian Institute of Geography and Statistics) website http://downloads.ibge.gov.br/downloads_geociencias.htm.

We hypothesized that *K* would be associated with environmental and demographic characteristics of the neighborhoods (Table 2). We found *K* associated with neighborhood size, but not with population density. Among the variables related to the production of breeding sites, only *% households with garbage in the streets* was associated and with less intensity, only to *aIMFA*, *% households with open sewage*. In addition, the variable *% households in streets without manhole* was associated negatively with *K* (Fig 6).

### Relationship between carrying capacity, mosquito abundance and capture rate

The mathematical relationship between the carrying capacity *K* and capture rate *α*, as a function of the number of adult mosquitoes in the equilibrium is:

$$\begin{array}{c} K=A^{*}\;{\left(\frac{1}{\frac{1}{\sigma_{3}}\Phi_{L}\Phi_{P}\;(\alpha \frac{T_{n}}{H_{n}}+\mu_{4})}-\Phi_{E}\right)}^{-1} \end{array}$$ (8)

where Φ_*E*_, Φ_*L*_, Φ_*P*_ are combinations of the life history parameters (see S1 Appendix for the derivation of this expression from the mathematical model).

According to this expression shows, *K* should increase linearly with the abundance of adult mosquitoes (what we found) but this relationship depends on a composite factor of life-history parameters and the trapping process. It is clear that the estimated *K* will vary if trap attractiveness varies (*α*), in other words, the estimated *K* is trap dependent.

## Discussion

Entomological surveillance is a key component of any dengue control program. Infestation indexes are collected to inform levels of attention and to identify hotspots for vector control activities. Understanding the temporal dynamics of *Ae. aegypti* and its response to environmental factors is important for the development of early-warning systems and identification of urban landscapes most associated with infestation. In this study, we have shown that a mechanistic model with temperature-dependent transition rates is able to capture the temporal dynamics of *Ae. aegypti* in a dengue endemic tropical city and allows the estimation of the mosquito’s carrying capacity. It is known that high temperature is a strong modulator of *Ae. aegypti* dynamics [34, 35], once it stimulates the fast development of larvae and the likelihood of adult emergence, as well its dispersion [36, 37]. Our results are in agreement with previous work [21] who applied the same model to ovitrap data in Rio de Janeiro, and supports the hypothesis that mosquito abundance responds to temperature even when annual temperature amplitudes are not high [6].

In this study, we have found carrying capacities for textitAe. aegypti varying from 1383 to 4143 across Vitoria’s neighborhoods. These values represents an approximation of the egg maximum capacity load of each neighborhood, once it also depends on the stated efficiency of the trapping system to sample the adult population. In other words, a more attractive trap would collect a larger sample of the flying population than a less attractive one and the number of collect mosquitoes will affect the estimated *K*. Resende et al. [30] showed that trap sensitivity (Positive MosquiTRAP Index) increased significantly with 8 traps per block in both high and low abundance areas, suggesting that higher densities of MosquiTRAPs may be required for monitoring *Ae. aegypti*.

In the literature, there are suggestions that *Ae. aegypti* carrying capacity should increase with human population density and the amount of breeding sites produced by poor managed garbage disposal, and water storage. In Vitória, we have found evidence for association of *K* with garbage in the streets. The negative association between the *% households in streets without manholes* and *K* could be explained by manholes functioning as oviposition sites in the urban landscape. The presence of immature *Ae. aegypti* in the drainage system without cleaning and maintenance is well understood [38, 39], being considered a potential breeding site by the Center for Disease Control and Prevention (CDC) [40]. In Brazil, it is commonly stated that most breeding sites are within the households and their owners should be responsible for their removal. Although we did not directly address this hypothesis, the results presented here suggest that the landscape outside the households also contributes to the carrying capacity of *Ae. aegypti*. Differences in receptivity conditions, as found in the neighborhoods of Vitória, are expected in an urban landscape. It is likely that even within neighborhoods, the short flight capacity of *Ae. aegypti* will favor the formation of small clusters of mosquitoes [41]. Therefore, improving sanitary conditions and public services to reduce mosquito density is an important control policy [42, 43] as well as defining and directing more intense efforts of vector control actions to the clusters of greater risk.

The entomological surveillance program implemented in Vitória operates with 2 to 149 traps per neighborhood, which is the spatial scale at which *IMFA* is calculated and control actions implemented. Our results have indicated that the estimation of carrying capacity is affected by the sampling effort (number of traps). Small neighborhoods, with small sampling effort, were found to have less precise trap data to be modeled. A closer inspection indicates a higher variability in *IMFA* as sample size decreases. Better than average goodness-of-fit has been only observed with 16 or more traps installed (Fig 5). Although this is not a study designed for sample size calculation, our results suggest that a minimum of 16 traps should be considered for monitoring a neighborhood. Otherwise, if less traps are delivered per area, than *IMFA* should be calculated with less spatial resolution.

There are some limitations in this study. The satellite data is only reliable on days with few clouds, and under perfect conditions, every 8 days. There is only one meteorological station in the city, at the airport. Cities with the geographical complexity of Vitoria should have meteorological surveillance integrated with their entomological surveillance. The microclimate variations within a city can be strong enough to offer different survival and reproduction conditions for invertebrates [44] such as *Ae. aegypti*. The model also present limitations, as it considers temperature as the only source of temporal variation. Rainfall and relative air humidity may be extra sources of variation that should be considered in future models. It is also necessary to further explore spatial dependences between neighborhoods. Finally, the parameters of the model, except for *K*, were fixed in values obtained from the literature, which do not necessarily reflect the biology of the mosquitoes in Vitória.

In summary, the trap based surveillance system employed in Vitória delivers infestation indices that are consistent with the patterns predicted by *Ae. aegypti* ecological models. This result supports the usefulness of the trap-based surveillance for guiding *Ae. aegypti* control programs, in combination with mathematical models that allow the estimation of the unobservable carrying capacity. For local assessments, the quality of the indices depended on the trap sample size. We recommend that *IMFA* indices should be calculated with at least 16 traps per neighborhood. From a modeling perspective, this study highlights the importance of mathematical models beyond their applications in ecology. Besides estimating latent (hidden) variables, once calibrated, models can be used to point out geographic areas and levels for the control of vector-borne diseases.

## Supporting information

S1 Table Convert data used for analysis. IMFA and temperature data.
(XLS)

S1 Appendix Equilibrium states and mathematical relationship between carrying capacity (*K*) and capture rate (*α*). (PDF)
