# Supplementary material for: Assessment of a trap based Aedes aegypti surveillance program using mathematical modeling
Source: PLoS One. 2018 Jan 5;13(1):e0190673. doi: 10.1371/journal.pone.0190673 (PMC5755894; doi:10.1371/journal.pone.0190673)
Supplement: S1 Appendix — (PDF) [file pone.0190673.s002.pdf]

S1 Appendix. Equilibrium states and mathematical relationship between Carrying capacity (K) and capture rate (α).

Raquel Martins Lana[1*], Maíra Moreira Morais[2], Tiago França Melo de Lima[3], Tiago Garcia de Senna Carneiro[4], Lucas Martins Stolerman[1], Jefferson Pereira Caldas dos Santos[5], José Joaquín Carvajal Cortés[6], Álvaro Eduardo Eiras[7], Cláudia Torres Codeço[1]

1 Programa de Computação Científica, PROCC/ Fiocruz, Rio de Janeiro - RJ, Brazil

2 Centro Universitário de Belo Horizonte (UNIBH), Belo Horizonte, Minas Gerais, Brasil

3 Departamento de Computação e Sistemas (DECSI), Instituto de Ciências Exatas e Aplicadas (ICEA), Universidade Federal de Ouro Preto (UFOP), João Monlevade, Minas Gerais, Brasil

4 Departamento de Computação, Universidade Federal de Ouro Preto, MG, Brazil

5 Programa de Pós-Graduação em Epidemiologia em Saúde Pública, Escola Nacional de Saúde Pública Sérgio Arouca (ESNP), Fundação Oswaldo Cruz (Fiocruz), Rio de Janeiro, Rio de Janeiro, Brasil

6 Laboratório de Doenças Parasitárias, Instituto Oswaldo Cruz/Fiocruz, Rio de Janeiro - RJ, Brazil

7 Laboratório de Ecologia Química de Insetos Vetores (Labeq), Departamento de Parasitologia Instituto de Ciências Biológicas (ICB), Universidade Federal de Minas Gerais, Belo Horizonte - MG, Brazil

Here we show all the steps for the mathematical derivation of the equilibrium states in our model, assuming a fixed temperature scenario. From this calculation, an exact formula relating the carrying capacity (K) and the capture rate (α) is obtained. It is worth noting that in the time-dependent case, the steady states would specific trajectories of the system variables given by the equations we derive in this section.

The equilibrium solutions, from now on denoted by the labels $\{E^*, L^*, P^*, A^*\}$ can be found by solving the following system

$$\begin{aligned}
\sigma_0 A^* \left[1 - \frac{E^*}{K}\right] - [\sigma_1 + \mu_1]\, E^* &= 0 \\
\sigma_1 E^* - [\sigma_2 + \mu_2]\, L^* &= 0 \\
\sigma_2 L^* - [\sigma_3 + \mu_3]\, P^* &= 0 \\
\sigma_3 P^* - \left[\alpha \frac{T_n}{H_n} + \mu_4\right] A^* &= 0
\end{aligned}$$

meaning that the derivatives of each component in the model is equal do zero. If we introduce the auxiliary variables

$$\Phi_E = \frac{\sigma_1 + \mu_1}{\sigma_0}, \quad \Phi_L = \frac{\sigma_2 + \mu_2}{\sigma_1}, \quad \Phi_P = \frac{\sigma_3 + \mu_3}{\sigma_2} \quad \text{and} \quad \Phi_A = \frac{1}{\sigma_3}\left(\alpha \frac{T_n}{H_n} + \mu_4\right),$$

then we can rewrite the algebraic system in the simpler form

$$A^* \left[1 - \frac{E^*}{K}\right] - \Phi_E E^* = 0 \tag{1a}$$

$$E^* = \Phi_L L^* \tag{1b}$$

$$L^* = \Phi_P P^* \tag{1c}$$

$$P^* = \Phi_A A^* \tag{1d}$$

Using equations 1b, 1c and 1d, we can write $E^* = \Phi_L \Phi_P \Phi_A A^*$ and by substituting in 1a, we arrive at

$$A^* \left[1 - \frac{\Phi_L \Phi_P \Phi_A A^*}{K} - \Phi_E \Phi_L \Phi_P \Phi_A\right] = 0.$$

The equilibrium states for the adult population are then given by $A^* \equiv 0$ (trivial state without any population) and

$$A^* = K\left(\frac{1}{\Phi_L \Phi_P \Phi_A} - \Phi_E\right) \tag{2}$$

The solutions for the remaining variables are found by substituting (2) in to the other equations. Then we can write

$$P^* = K\left(\frac{1}{\Phi_L\Phi_P} - \Phi_E\Phi_A\right), \quad L^* = K\left(\frac{1}{\Phi_L} - \Phi_E\Phi_P\Phi_A\right) \quad \text{and}$$

and $E^* = K\ (1 - \Phi_E\Phi_L\Phi_P\Phi_A)$

From equation (2), by substituting the expression of Φ_A, we also obtain the mathematical relationship between the carrying capacity K and capture rate α, as a function of the number of adult mosquitoes in the equilibrium:

$$K = A^*\left(\frac{1}{\frac{1}{\sigma_3}\Phi_L\Phi_P\left(\alpha\frac{T_n}{H_n} + \mu_4\right)} - \Phi_E\right)^{-1}$$
